# Joint frailty modeling of time-to-event data to elicit the evolution pathway of events: a generalized linear mixed model approach

**DOI:** 10.1093/biostatistics/kxab037

**Published:** 2021-11-09

**Authors:** Shu Kay Ng, Richard Tawiah, Geoffrey J Mclachlan, Vinod Gopalan

**Affiliations:** School of Medicine and Dentistry, Menzies Health Institute Queensland, Griffith University, Nathan, QLD 4111, Australia; School of Mathematics and Statistics, University of Melbourne, Parkville, VIC 3010, Australia; Department of Mathematics, University of Queensland, St. Lucia, QLD 4072, Australia; School of Medicine and Dentistry, Menzies Health Institute Queensland, Griffith University, Southport, QLD 4222, Australia

**Keywords:** Cancer registry data, Generalized linear mixed models, Informative censoring, Mean residual life, Multimorbidity, Secondary primary cancer

## Abstract

Multimorbidity constitutes a serious challenge on the healthcare systems in the world, due to its association with poorer health-related outcomes, more complex clinical management, increases in health service utilization and costs, but a decrease in productivity. However, to date, most evidence on multimorbidity is derived from cross-sectional studies that have limited capacity to understand the pathway of multimorbid conditions. In this article, we present an innovative perspective on analyzing longitudinal data within a statistical framework of survival analysis of time-to-event recurrent data. The proposed methodology is based on a joint frailty modeling approach with multivariate random effects to account for the heterogeneous risk of failure and the presence of informative censoring due to a terminal event. We develop a generalized linear mixed model method for the efficient estimation of parameters. We demonstrate the capacity of our approach using a real cancer registry data set on the multimorbidity of melanoma patients and document the relative performance of the proposed joint frailty model to the natural competitor of a standard frailty model via extensive simulation studies. Our new approach is timely to advance evidence-based knowledge to address increasingly complex needs related to multimorbidity and develop interventions that are most effective and viable to better help a large number of individuals with multiple conditions.

## 1. Introduction

A recent editorial published in *Lancet* and the UK Academy of Medical Sciences in 2018 has identified multimorbidity as a priority for global research in health sciences (Lancet editorial, 2018; Academy of Medical Science, 2018). Multimorbidity, defined as the coexistence of two or more chronic conditions within a single patient (van den Akker *and others,* 1996), constitutes a serious burden and challenge on individuals and healthcare systems in many countries because of its association with poorer health-related outcomes, lower quality of life, increases in health service utilization and costs, and a decrease in productivity (Di Angelantonio *and others,* 2015; Ng *and others,* 2019b; Pearson-Stuttard *and others,* 2019). However, to date, most evidence on multimorbidity is derived from cross-sectional studies, focusing on identifying groups of multimorbid health conditions (Marengoni *and others,* 2009; Ng *and others,* 2012, 2018) or groups of individuals with different multimorbidity patterns (Ng, 2015; Ng *and others,* 2019a) via cluster analysis. These studies have had limited capacity to understand the pathway of multimorbid conditions across the lifespan and possible shared biologic processes in the etiology of specific diseases and the effects of multimorbidity on an individual’s intrinsic capacity and functional ability (Prados-Torres *and others,* 2014; Valderas *and others,* 2009). Research on longitudinal multimorbidity data has been scarce or has been limited by being exploratory in nature (Ashworth *and others,* 2019; Ruel *and others,* 2014; Vos *and others,* 2015).

In this article, we take an innovative perspective on analyzing longitudinal multimorbidity data within a statistical framework of survival analysis, with an attempt to open a new way to advance the knowledge on the onset and the evolution pathway of multimorbidity. In survival analysis of longitudinal time-to-event data, there are a number of formidable challenges due to the heterogeneity in individual’s risk of failure, the loss of the appealing cancelation property in the estimation process for the parameters in Cox regression models, and the presence of “informative censoring” due to a “terminal” event; see Ng *and others* (2019b) for an overview on these issues. Here, we develop a new joint frailty modeling approach via a generalized linear mixed model (GLMM) formulation to address the aforementioned methodological barriers for efficient estimation of model parameters. The advantages of frailty models are their capacity to account for heterogeneity in individual responses using (multivariate) random effects. The proposed estimation method extends the GLMM approach of McGilchrist (1994) to joint frailty models for survival analysis of censored data with recurrent and terminal events. The original work of McGilchrist (1994) extended the best linear unbiased prediction (BLUP) method for linear mixed models (LMMs) to a GLMM setting (with additional random effect terms in the linear predictor) through the residual maximum likelihood (REML) estimation procedures.

Novelty of this article lies in how the multivariate random effects are set up in the joint frailty model to account for the heterogeneous risk of multimorbidity and informative censoring such that time to recurrent events (multimorbidity) and time to death observed on the same subject are correlated. Existing joint frailty modeling methods to handle informative censoring have been implemented by assuming a shared patient-specific frailty term under an integrated likelihood approach or a Bayesian setting (Huang and Wolfe, 2002; Król *and others,* 2017; Liu *and others,* 2004; Paulon *and others,* 2020; Rizopoulos, 2012) or a different procedure for parameter estimation (e.g., the hierarchical likelihood method in Christian *and others* (2016) in a competing-risk context). In our approach, we consider a more general model that allows for two correlated random effects to jointly model the dependence between the hazard rates of recurrent events and the terminal event. In contrast to shared frailty models, our model sets up two sets of random effects to account for intrasubject correlation of multivariate recurrent event times and individual differences in mortality hazard rate. Some advantages of the proposed method include its flexibility to distinguish the origin of dependence and allow for a positive or negative association between recurrent and terminal events, as well as the use of GLMM to avoid the needs for intractable high-dimensional integration involved in the marginal likelihood methods or time-consuming Monte Carlo approximations in the E-step for maximum likelihood estimation. Our previous work focuses on mixture cure models for recurrent event data with a terminal event (Tawiah *and others,* 2020a) or clustered recurrent event data without a terminal event (Tawiah *and others,* 2020b). While both frailty models adopt a GLMM formulation for parameter estimation, a distinctive feature of the joint frailty model proposed in this article is the focus of the modeling of the distributions of multiple failure times without cure fraction. This model specification is particularly significant for multimorbidity research as the risk of co-occurrence of other health conditions is not generally reduced with time to justify the existence of “long-term survivors.” With this model specification and the proposed GLMM formulation, there is an advantage that the unknown baseline hazard functions can be canceled out from the partial likelihood and such an approach thus preserves the appealing cancelation property in which the baseline hazard functions are not involved in the estimation of regression coefficients and can be unspecified, making the estimation procedures relatively efficient.

We provide an overview of the proposed joint frailty model and an application of the model to a real cancer registry data set on the multimorbidity of melanoma patients in Australia. We then document the performance of the joint frailty model via extensive simulation studies. These show that the proposed model performs well in a wide variety of contexts, and it is superior to the natural competitor of a standard frailty model, which does not take into consideration informative censoring due to a terminal event.

## 2. Methods

Suppose we follow up $M$ independent patients from the study onset and observe the gap times of events experienced by each patient. The event of interest corresponds to a recurrent episode, such as tumor relapses or in this study multimorbidity of health conditions. Denote $T_{jk}^R$ the gap times of the recurrent event and $T_j^D$ the gap time from the last recurrent event to death, which are both subject to right censoring ($j=1,\ldots,M; \ k=1,2,\ldots$). Here, death is considered as a terminal event, creating a mechanism of informative censoring where the recurrent and terminal events are correlated in contrast to a typical assumption of independent censoring in survival analysis; see, for example, Huang and Wolfe (2002), Liu *and others* (2004), Tawiah *and others* (2020a). That is, censoring due to death is informative for failure due to the recurrent event. On the other hand, the gap censoring time from the last event to the end of the study (also known as “administrative censoring”), denoted by $C_j$, is noninformative (Huang and Wolfe, 2002). Let $T_{jk}=\min(T_{jk}^R,T_j^D,C_j)$ and define the indicator variables for a recurrent event and death, respectively, $\delta_{jk}^R=1$ if $T_{jk}=T_{jk}^R$, $\delta_j^D=1$ if $T_{jk}=T_j^D$, and both to be 0 if censored (i.e., $T_{jk}=C_j$). The observed data on the $j$th patient is thus given by $O_j= \{(t_{jk},\delta_{jk}^R,\delta_j^D,{\boldsymbol{x}}_j),j=1,\ldots,M; k=1,\ldots,n_j \}$, where ${\boldsymbol{X}}_j=(X_{j1},\ldots,X_{jp})^T$ is a $p$-dimensional vector of risk variables and $n_j$ denotes the number of observed gap times for the $j$th patient. The superscript $T$ denotes vector transpose. Overall, there are $\sum_{j=1}^M n_j = N$ observations. In contrast to the use of a shared frailty term (Huang and Wolfe, 2002), we denote ${\boldsymbol{u}}=(u_1,\ldots,u_M)^T$ and ${\boldsymbol{v}}=(v_1,\ldots,v_M)^T$ be the random vectors of $u_j$ and $v_j$ that represent the frailty for the $j$th patient to account for intra-subject correlation of multivariate recurrent event times and individual differences in mortality hazard rate for the death time, respectively. Assuming that ${\boldsymbol{q}}=({\boldsymbol{u}}^T,{\boldsymbol{v}}^T)^T$ follows a multivariate normal distribution $N({\boldsymbol{0}},{\boldsymbol{\Sigma}})$, we consider the variance–covariance matrix as ${\boldsymbol{\Sigma}}=\Gamma \otimes I_{M}$, where

(2.1) \begin{equation*} \Gamma=\begin{bmatrix} \theta_{u}^{2} & \rho \theta_{u}\theta_{v}\\ \rho \theta_{u}\theta_{v} & \theta_{v}^{2} \\ \end{bmatrix}\!, \label{eq:1} \end{equation*}

$I_M$ is an identity matrix with dimension $M$, and $\otimes$ denotes the Kronecker product of two matrices. In (2.1), $\theta_{u}^{2}$ and $\theta_{v}^{2}$ quantify the heterogeneity in the unobserved random (frailty) effects for the hazard rates of recurrent events and death, respectively, whereas a correlation parameter $\rho$ is adopted to model the dependence between ${\boldsymbol{u}}$ and ${\boldsymbol{v}}$; see, for example, Tawiah *and others* (2020a).

Under the Cox proportional hazards (PH) model, the hazard functions for recurrent events and death are given by

(2.2) \begin{eqnarray*} && h_{R}\left(t_{jk}^R;{\boldsymbol{x}}_{j} \right)=h_{R0}\left(t_{jk}^{R} \right)\exp\left( \eta_{jk} \right), \nonumber \\ && h_{D}\left(t_{j}^D;{\boldsymbol{x}}_{j} \right)=h_{D0}\left(t_{j}^D \right)\exp\left( \zeta_{j} \right), \label{eq:2} \end{eqnarray*}

where $h_{R0}(t_{jk}^R )$ and $h_{D0}(t_{j}^D)$ are the baseline hazard functions, $\eta_{jk}$ and $\zeta_{j}$ are the linear predictors for recurrent events and death, respectively, which relate to the risk covariates ${\boldsymbol{x}}_j$ as:

(2.3) \begin{equation*} \eta_{jk} ={\boldsymbol{x}}^{T}_{j}{\boldsymbol{\beta}}+u_{j} \hspace{0.5cm} \mbox{and} \hspace{0.5cm} \zeta_{j} ={\boldsymbol{x}}^{T}_{j}{\boldsymbol{\gamma}}+v_{j}, \label{eq:3} \end{equation*}

where ${\boldsymbol{\beta}}$ and ${\boldsymbol{\gamma}}$ are the vectors of fixed-effect regression coefficients for recurrent events and death, respectively. A positive value of coefficients in ${\boldsymbol{\beta}}$ or ${\boldsymbol{\gamma}}$ implies a higher risk of failure associated with the risk covariates. In (2.3), we denote the covariates be a $p$-dimensional vector ${\boldsymbol{x}}_j$, but selections of covariates entering the model can be different for the recurrent events and death.

### 2.1. Joint modeling via GLMM

We develop a GLMM method for the joint modeling of $h_{R}\left(t_{jk}^R;{\boldsymbol{x}}_{j} \right)$ and $h_{D}\left(t_{j}^D;{\boldsymbol{x}}_{j} \right)$ with random effects terms in $\eta_{jk}$ and $\zeta_{j}$; see (2.2) and (2.3). With this GLMM formulation, multivariate random effects are added to the linear predictors and the corresponding partial log-likelihood of failure times with the random effects conditionally fixed can be constructed. Besides its capacity to assess and estimate the heterogeneity in individual risk of failure in recurrent events or death, another appealing aspect of the GLMM approach is to retain the cancelation property where the unknown baseline hazard functions are eliminated in the estimation process for the regression parameters, leading to more efficient estimation procedures relatively and that the baseline hazard functions can be unspecified (Ng *and others,* 2019b; Yau, 2001).

Denote ${\boldsymbol{\Omega}}=({\boldsymbol{\beta}}^T,{\boldsymbol{\gamma}}^T,{\boldsymbol{u}}^T,{\boldsymbol{v}}^T)^T$. The GLMM method starts with developing BLUP estimators for ${\boldsymbol{\Omega}}$ that maximize the sum of two components:

(2.4) \begin{eqnarray*} l_1 &=& \mbox{ Joint partial log-likelihood of failure times taking } {\boldsymbol{u}} \mbox{ and } {\boldsymbol{v}} \mbox{ fixed}, \nonumber \\ l_2 &=& -\frac{1}{2}\left\lbrace M \log (2 \pi|{\boldsymbol{\Sigma}}|)+ ({\boldsymbol{u}}^T,{\boldsymbol{v}}^T) {\boldsymbol{\Sigma}}^{-1}({\boldsymbol{u}}^T,{\boldsymbol{v}}^T)^T\right\rbrace\!, \qquad \label{eq:4} \end{eqnarray*}

where $l_2$ is the logarithm of the joint probability density function of random effects ${\boldsymbol{u}}$ and ${\boldsymbol{v}}$. With (2.2) and (2.3), the likelihood contribution for the $j$th individual is given by

$$
\prod_{k=1}^{n_j} \left\{ h_{R0}\left(t_{jk}^{R} \right)\exp\left( \eta_{jk} \right)\right\} ^{\delta_{jk}^R}
\exp \left\{-H_{R0}\left(t_{jk}^{R} \right)\exp\left( \eta_{jk} \right)\right\} \times
\left\{h_{D0}\left(t_{j}^{D} \right)\exp\left( \zeta_{j} \right)\right\}^{\delta_{j}^D}
\exp \left\{-H_{D0}\left(t_{j}^{D} \right)\exp\left( \zeta_{j} \right)\right\}\!,$$

from which the partial log-likelihood $l_1$ can be derived. In the above formula, $H_{R0}(t_{jk}^R )$ and $H_{D0}(t_{j}^D)$ are the baseline cumulative hazard functions for recurrent events and death, respectively. Thus, the parameters for recurrent events are jointly estimated with death, accounting for the correlation between the frailty terms ${\boldsymbol{u}}$ and ${\boldsymbol{v}}$. As the last event is either death $(t_{jk}= t_j^D)$ or censored $(t_{jk}=C_j)$, it implies that when $\delta_{j}^D=1$ indicating death, there are no more contribution terms from the same subject on recurrent events. Practically, $l_1$ is identifiable if the data $O_j \ (j=1,\ldots,M)$ provide sufficient information that distinguishes the censoring mechanisms between $\delta_{jk}^R$ and $\delta_j^D$ and that the recurrent event times and the death times are assumed to be independent conditional on ${\boldsymbol{u}}$ and ${\boldsymbol{v}}$; see Tawiah *and others* (2020a).

To write down the partial log-likelihood $l_1$, the recurrent event gap/censoring times and the death/censoring times are arranged in increasing order, with their corresponding linear predictors and indicator variables representing failure or censoring. The respective distinct reordered uncensored times are denoted by $t_{i1},\ldots,t_{iK_i}$ for $i=R$ (recurrent events) and $i=D$ (death). Assuming a step function with discontinuities at each observed failure time for the baseline hazard functions $h_{i0}(t_i)$ for recurrent events $(i=R)$ or death $(i=D)$, it can be shown from Breslow (1974) that the joint partial log-likelihood for the Cox PH model, conditional on fixed random effects, is given by:

(2.5) \begin{equation*} l_{1} = \sum_{r=1}^{K_R}\left\lbrace \eta_{r}- m_r^R \log \sum_{l\in R(t_{Rr})} \exp(\eta_{l})\right\rbrace + \sum_{r=1}^{K_D}\left\lbrace \zeta_{r}- m_r^D \log \sum_{l\in R(t_{Dr})} \exp(\zeta_{l})\right\rbrace\!, \label{eq:5} \end{equation*}

where $m_r^i$ is the number of uncensored failures at $t_{ir}$ ($i=R$ or $i=D$), $\eta_r$ and $\zeta_r$ represent the sum of linear predictors over the $m_r^R$ and $m_r^D$ tied observed failures, respectively, and $R(t_{Rr})$ and $R(t_{Dr})$ are the risk sets at time $t_{Rr}$ and $t_{Dr}$ corresponding to the gap times of the recurrent events and the death times, respectively. Based on the BLUP estimates, the approximate REML estimate of the variance component parameters in ${\boldsymbol{\Sigma}}$, denoted as ${\boldsymbol{\Phi}}=(\theta_u^2,\theta_v^2,\rho)^T$, is then obtained by solving the equation of the first-order derivative of the REML log likelihood with respect to ${\boldsymbol{\Phi}}$ (Yau, 2001). The asymptotic variances of the estimates in ${\boldsymbol{\beta}}$, ${\boldsymbol{\gamma}}$, and ${\boldsymbol{\Phi}}$ are then obtained via the second derivative of $l=l_1+l_2$ with respect to the conformal partition of ${\boldsymbol{\beta}}|{\boldsymbol{\gamma}}|{\boldsymbol{u}}|{\boldsymbol{v}}$, as outlined in Section S1 of Supplementary material available at *Biostatistics* online.

### 2.2. Algorithm

The Newton–Raphson iterative procedure is adopted to update the BLUP estimates by finding the solution that maximizes (2.4). Let ${\boldsymbol{X}}_1,{\boldsymbol{X}}_2,{\boldsymbol{Z}}_1$, and ${\boldsymbol{Z}}_2$ denote the design matrices of ${\boldsymbol{\beta}},{\boldsymbol{\gamma}},{\boldsymbol{u}}$, and ${\boldsymbol{v}}$, respectively, and ${\boldsymbol{G}}^{-1}$ denote the inverse of the information matrix corresponding to $l$. In the $k$th iteration, we have:

(2.6) \begin{equation*} \begin{bmatrix} {\boldsymbol{\beta}}^{(k)}\\ {\boldsymbol{\gamma}}^{(k)}\\ {\boldsymbol{u}}^{(k)}\\ {\boldsymbol{v}}^{(k)}\\ \end{bmatrix}= \begin{bmatrix} {\boldsymbol{\beta}}^{(k-1)}\\ {\boldsymbol{\gamma}}^{(k-1)}\\ {\boldsymbol{u}}^{(k-1)}\\ {\boldsymbol{v}}^{(k-1)}\\ \end{bmatrix}+ {\boldsymbol{G}}^{-1} \begin{bmatrix} \partial l/\partial{\boldsymbol{\beta}}\\ \partial l/\partial{\boldsymbol{\gamma}}\\ \partial l/\partial {\boldsymbol{u}}\\ \partial l/\partial {\boldsymbol{v}}\\ \end{bmatrix}\!, \label{eq:8} \end{equation*}

where

$$
\frac{\partial l}{\partial {\boldsymbol{\beta}}}={\boldsymbol{X}}_{1}^{T}\frac{\partial l_{1}}{\partial \eta}; \
\frac{\partial l}{\partial {\boldsymbol{\gamma}}}={\boldsymbol{X}}_{2}^{T}\frac{\partial l_{1}}{\partial \zeta}; \
\frac{\partial l}{\partial {\boldsymbol{u}}}={\boldsymbol{Z}}_{1}^T\frac{\partial l_{1}}{\partial \eta}-\frac{{\boldsymbol{u}}\theta_{v}^2- {\boldsymbol{v}}\rho\theta_{u}\theta_{v}}{\theta_{u}^2 \theta_{v}^2\left(1-\rho^2 \right) }; \
\frac{\partial l}{\partial {\boldsymbol{v}}}={\boldsymbol{Z}}_{2}^T\frac{\partial l_{1}}{\partial \zeta}-\frac{{\boldsymbol{v}}\theta_{u}^2-{\boldsymbol{u}}\rho\theta_{u}\theta_{v}}{\theta_{u}^2 \theta_{v}^2\left(1-\rho^2 \right)};
$$

see Section S1 of Supplementary material available at *Biostatistics* online for the block matrices of ${\boldsymbol{G}}^{-1}$ and the derivation of $\partial l_{1}/\partial \eta$ and $\partial l_{1}/\partial \zeta$.

By solving the equation of the first-order derivative of the REML log likelihood with respect to ${\boldsymbol{\Phi}}$, it follows that an update of the approximate REML estimators of ${\boldsymbol{\Phi}}$ is given by:

(2.7) \begin{equation*} \theta_{u}^{2}=\frac{1}{M}\Im_{1}, \hspace{0.2cm} \rho=\frac{1}{\sqrt{\Im_{1}\Im_{3}}}\Im_{2}, \hspace{0.2cm} \mbox{and} \hspace{0.2cm} \theta_{v}^{2}=\frac{1}{M}\Im_{3}, \label{eq:14} \end{equation*}

where $\Im_{1}=\mbox{tr}\left\lbrace K_{1} \left( {\boldsymbol{B}}_{q,q}+ {\boldsymbol{q}}{\boldsymbol{q}}^{T} \right) \right\rbrace\!, \ \Im_{2}=\mbox{tr}\left\lbrace K_{2} \left( {\boldsymbol{B}}_{q,q}+ {\boldsymbol{q}}{\boldsymbol{q}}^{T} \right) \right\rbrace /2, \ \Im_{3}=\mbox{tr}\left\lbrace K_{3} \left( {\boldsymbol{B}}_{q,q}+ {\boldsymbol{q}}{\boldsymbol{q}}^{T} \right) \right\rbrace$ are calculated based on the current estimates of ${\boldsymbol{\Omega}}$. Here, $\mbox{tr}$ denotes the trace of a matrix, ${\boldsymbol{B}}_{q,q}$ is the block matrix of ${\boldsymbol{G}}^{-1}$ corresponding to the random effects ${\boldsymbol{q}}$, and the block matrices $K_{1}, K_{2}$ and $K_{3}$ are defined as:

\begin{equation*} K_{1}=\begin{bmatrix} I_{M} & 0 \\ 0 & 0\\ \end{bmatrix}\!, \hspace{0.3cm} K_{2}= \begin{bmatrix} 0 & I_{M} \\ I_{M} & 0\\ \end{bmatrix}\!, \hspace{0.3cm} \mbox{and} \hspace{0.3cm} K_{3}= \begin{bmatrix} 0 & 0 \\ 0 & I_{M}\\ \end{bmatrix};\end{equation*}

see Section S1 of Supplementary material available at *Biostatistics* online for the formulation of ${\boldsymbol{B}}_{q,q}$.

The following summarizes the computational procedure for the proposed joint modeling of recurrent and death times within the GLMM framework:

1. Set the initial values of ${\boldsymbol{\Omega}}^{(0)}$ (corresponding to ${\boldsymbol{\beta}}^{(0)}$, ${\boldsymbol{\gamma}}^{(0)}$, ${\boldsymbol{u}}^{(0)}$, and ${\boldsymbol{v}}^{(0)}$) to zero and the initial values of ${\boldsymbol{\Phi}}^{(0)}$ (corresponding to $\theta_u^{2(0)}$, $\theta_v^{2(0)}$, and $\rho^{(0)}$) to relatively small values.
2. Given the current estimates of ${\boldsymbol{\Omega}}$ and ${\boldsymbol{\Phi}}$, update the estimates of ${\boldsymbol{\Omega}}$ based on the BLUP method (2.6) until convergence.
3. Update the variance component parameters in ${\boldsymbol{\Phi}}$ based on the REML approach via (2.7).
4. Repeat Steps 2 and 3 until convergence.
5. Calculate the standard errors of estimates in ${\boldsymbol{\Omega}}$ using the block matrices ${\boldsymbol{B}}_{\beta,\beta}$ and ${\boldsymbol{B}}_{\gamma,\gamma}$ in Equation (S1.1) of Supplementary material available at *Biostatistics* online, respectively.
6. Calculate the standard errors of estimates in ${\boldsymbol{\Phi}}$ using Equation (S1.5) of Supplementary material available at *Biostatistics* online, corresponding to the inversion of the REML information matrix.

As manipulations of large matrices are involved in the above procedure, the computational complexity and cost of the proposed method are high. For example, update based on (2.6) in Step 2 appears in every iteration and involves the computation of $2(p+M) \times 2(p+M)$ matrices ${\boldsymbol{G}}$ and ${\boldsymbol{G}}^{-1}$. The time for calculating the standard errors in Step 6 is also high, but it is needed only once after the final estimates in ${\boldsymbol{\Phi}}$ are obtained. Furthermore, prediction intervals of the frailties can be obtained using the empirical Bayes (EB) method to estimate the variances of the random effects. The point estimates of the random effects and their EB prediction interval using a normal approximation can be plotted together to provide inferences concerning heterogeneity of the individual frailties among the subjects; see, for example, Vaida and Xu (2000).

### 2.3. Prediction of overall survival and mean residual life functions

Given $\hat{{\boldsymbol{\Omega}}}$, the baseline survival functions corresponding to the baseline hazard functions for recurrent events and death, respectively, $h_{R0}(t_{jk}^R )$ and $h_{D0}(t_{j}^D)$ can be estimated using the Breslow-type estimator (Breslow, 1974). That is,

(2.8) \begin{eqnarray*} &&\hat{S}_{R0}(t) = \exp \left\lbrace - \sum_{r: t_{Rr} \leq t} \left( \frac{m_r^R}{\sum_{l\in R(t_{Rr})} \exp(\eta_{l})} \right) \right\rbrace\!, \nonumber \\ &&\hat{S}_{D0}(t) = \exp \left\lbrace - \sum_{r: t_{Dr} \leq t} \left( \frac{m_r^D}{\sum_{l\in R(t_{Dr})} \exp(\zeta_{l})} \right) \right\rbrace\!, \quad \label{eq:15} \end{eqnarray*}

where the sums indexed by $r$ are taken over all $t_{ir} \leq t$, corresponding to the distinct reordered uncensored times for $i=R$ (recurrent events) or $i=D$ (death), respectively. In applications where the largest observed gap time is censored, the tail of $S_{i0}(t)$ ($i=R$ or $i=D$) may be estimated using the exponential-tail completion method; see Peng (2003); Tawiah *and others* (2020b). That is, $\hat{S}_{i0}(t)=\exp(- \hat{\lambda}_i t)$ for $t > t_{hi}$, where $t_{hi}$ is the largest uncensored gap time ($i=R$ or $i=D$) and $\hat{\lambda}= - \log \{ \hat{S}_{i0}(t_{hi}) \} / t_{hi}$.

With the Cox PH model (2.2), a prediction of the overall survival function at time $t$ for a given subject with risk variables ${\boldsymbol{X}}$ and zero subject effect (frailty) can be obtained as

(2.9) \begin{equation*} \hat{S}(t;{\boldsymbol{X}})=\hat{S}_{R0}(t)^{\exp({\boldsymbol{X}}^T\hat{{\boldsymbol{\beta}}})} \hat{S}_{D0}(t)^{\exp({\boldsymbol{X}}^T \hat{{\boldsymbol{\gamma}}})}, \label{eq:16} \end{equation*}

which summarizes subject-specific failure risk in terms of the probability of survival (free from recurrent event or death) at any time point $t$. A popular alternative to the survival function (2.9) is the mean residual life (MRL) function which summarizes failure risk in terms of the remaining life expectancy (i.e., in a unit of time rather than a probability). The concept of the MRL has been widely used in operational research, reliability, and statistics (Si *and others,* 2011). For a nonnegative failure time $T$ with finite expectation, the MRL function at time $t$ for a subject with ${\boldsymbol{X}}$ and zero subject effect is given by

(2.10) \begin{equation*} m(t;{\boldsymbol{X}})=E(T-t|T>t;{\boldsymbol{X}})=\frac{\int_t^{\infty} S(s;{\boldsymbol{X}}) ds}{S(t;{\boldsymbol{X}})}, \label{eq:17} \end{equation*}

providing the information on remaining subject-specific expected lifetime given survival up to time $t$ (Alvarez-Iglesias *and others,* 2015; Sun and Zhang, 2009). In this study, the MRL function is predicted by replacing $S(t;{\boldsymbol{X}})$ in (2.10) by $\hat{S}(t;{\boldsymbol{X}})$ from (2.9) which is obtained based on the PH assumption, a widely used and well-established approach in survival analysis. That is, (2.10) does not imply proportionality in the MRL functions. Alternatively, Chen *and others* (2005), Huang *and others* (2019), and Sun and Zhang (2009) developed “proportional MRL models,” which give a different interpretation of the covariate effect compared to (2.10). Furthermore, (2.9) and (2.10) represent the predicted survival and MRL functions over time for specific subjects with zero frailty, which will be different from the “population-averaged” values estimated using a marginal approach. However, the latter requires integration of the conditional functions over the frailty distribution, where the integrals may be approximated with the use of Gauss–Hermite quadrature method. Also, we adopt a subject-specific approach because this approach does not rely on the robustness of the estimation of the variance–covariance matrix $\Sigma$. Comparison studies of population-averaged survival and MRL functions and their interpretation are worth investigating in the future.

## 3. Results

### 3.1. A real example

The Queensland melanoma cancer registry data identify melanoma patients diagnosed and treated at Queensland hospitals in Australia in 2005 with follow-up until 2015. In this study, the Queensland melanoma cancer registry data were retrieved for melanoma patients aged $>50$ years, diagnosed and treated in Queensland in 2005. The occurrence of secondary primary cancer other than melanoma amongst this population during the follow-up period up to December 2015 was identified using International Classification of Diseases for Oncology (ICD-O) codes. Demographic and clinical information included age at diagnosis (relative to the age of 50 years), gender, country of birth (Overseas versus Australian-born), remoteness of residence at diagnosis (Outer regional/Remote/Very remote versus Major city/Inner regional), melanoma site (skin of trunk or skin of upper/lower limbs versus head/neck), lesion thickness ($>1$ mm versus $\le 1$ mm), and ulceration (presence versus absence). The data set consists of 1624 observations from 1399 patients (the number of secondary primary cancer other than melanoma ranges 0–2, with a mean of 0.164). There are 491 deaths, corresponding to a censoring proportion of 64.9% for $\delta_j^D$. The results of applying the proposed joint frailty model are presented in Table 1, along with those using a standard frailty model separately for multimorbidity and death events. The “multimorbidity” data were fitted via the GLMM without the assumption of informative censoring for comparison (the coxme package in R developed by Therneau (2018) produced the same conclusion for the standard frailty model), whereas the “death” data were fitted using the coxme package.

**Table 1.** Results of fitting the proposed joint frailty model and separate standard frailty models to the melanoma cancer registry data

	Parameter	Joint frailty model Hazard ratio (95% CI)	Frailty model$^\dagger$ Hazard ratio (95% CI)
Multimorbidity	Age	1.038 (1.022–1.054)	1.026 (1.012–1.041)
	Male	1.964 (1.367–2.822)	1.905 (1.376–2.637)
	Remoteness	0.613 (0.381–0.984)	0.622 (0.406–0.952)
	Melanoma site		
	Trunk	0.900 (0.560–1.447)	0.896 (0.590–1.360)
	Upper/lower limbs	1.012 (0.647–1.582)	1.090 (0.736–1.613)
	Head/Neck	Reference	Reference
	Thickness $>1$ mm	0.925 (0.615–1.391)	0.819 (0.569–1.179)
	Ulceration	2.683 (1.644–4.380)	2.022 (1.311–3.118)
Death	Age	1.113 (1.098–1.128)	1.105 (1.093–1.117)
	Male	2.022 (1.522–2.686)	1.685 (1.338–2.123)
	Remoteness	0.819 (0.580–1.156)	0.919 (0.695–1.216)
	Melanoma site		
	Trunk	0.753 (0.531–1.067)	0.825 (0.625–1.090)
	Upper/lower limbs	0.643 (0.465–0.891)	0.662 (0.512–0.856)
	Head/Neck	Reference	Reference
	Thickness $>1$ mm	1.799 (1.327–2.437)	1.781 (1.398–2.269)
	Ulceration	2.487 (1.748–3.539)	2.159 (1.645–2.834)
Patient frailty	$\theta_u^2$	1.978 (1.735–2.221)	0.687 (0.224–1.150)
	$\theta_v^2$	1.968 (1.725–2.211)	0.442
	$\rho$	0.964 (0.958–0.970)	n.a.

$^\dagger$ Standard frailty model applies separately to multimorbidity and death.

With the joint frailty model, it can be seen from Table 1 that older, male patients are associated with an increased risk for multimorbidity of a secondary cancer (HR = 1.038 per year older, 95% CI = 1.022–1.054, $p \ <$ 0.001; HR = 1.964 for male, 95% CI = 1.367–2.822, $p \ <$ 0.001) and death (HR = 1.113 per year older, 95% CI = 1.098–1.128, $p \ <$ 0.001; HR = 2.022 for male, 95% CI = 1.522–2.686, $p \ <$ 0.001). Patients with ulceration also have increased risks for multimorbidity (HR = 2.683, 95% CI = 1.644–4.380, $p \ <$ 0.001) as well as death (HR = 2.487, 95% CI = 1.748–3.539, $p \ <$ 0.001). Compared to patients living in major city or inner regional areas, those patients who are living in outer regional, remote, or very remote areas have a lower hazard risk for multimorbidity (HR = 0.613, 95% CI = 0.381–0.984, $p$ = 0.043) but a nonsignificant effect for risk of death. On the other hand, patients with lesion thickness $>$ 1 mm have a negligible effect in risk of multimorbidity but a higher risk for death (HR = 1.799, 95% CI = 1.327–2.437, $p \ <$ 0.001). Furthermore, patients with melanoma at skin of upper or lower limbs have a reduced risk of death (HR = 0.643, 95% CI = 0.465–0.891, $p$ = 0.008) compared to those with melanoma at the skin of head or neck. The estimates of the frailty variance parameters $\theta_u^2$, $\theta_v^2$, and $\rho$ are large and significant, indicating that there are meaningful heterogeneity in an individual’s risk of failure and positive dependence between the hazard rate of multimorbidity and death. The latter implies that the incidence of comorbid cancer is associated with poor survival outcomes with an increased risk of death.

While the results from separate standard frailty models given in Table 1 are comparable with those from the proposed joint frailty model, some slight differences can be observed. In particular, the estimated effects for patients with ulceration are smaller in separate frailty models and the estimates of the frailty variance parameters $\theta_u^2$ and $\theta_v^2$ are considerably smaller (about one-fifth to one-third of those in the joint frailty model). In practice, a major drawback of standard frailty models is the lack of information regarding death as a terminal event and its adjustment in the survival analysis; see also further comparisons between the joint frailty model and separate standard frailty models via simulations in the next subsection.

**Fig. 1.**
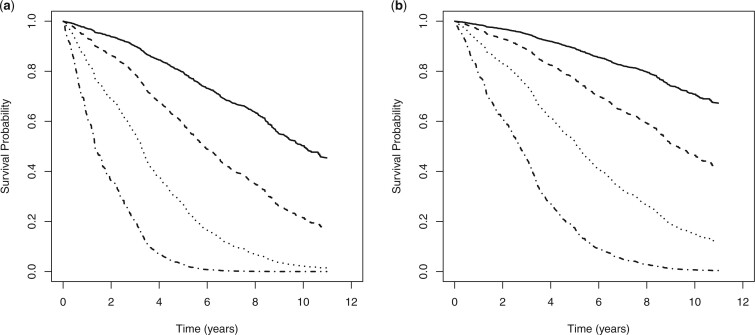
Predicted survival curves by age for (a) male patients and (b) female patients, with zero subject effect (age at diagnosis: 50 years [solid line]; 60 years [dashed line]; 70 years [dotted line]; 80 years [dashed-dotted line]).

**Fig. 2.**
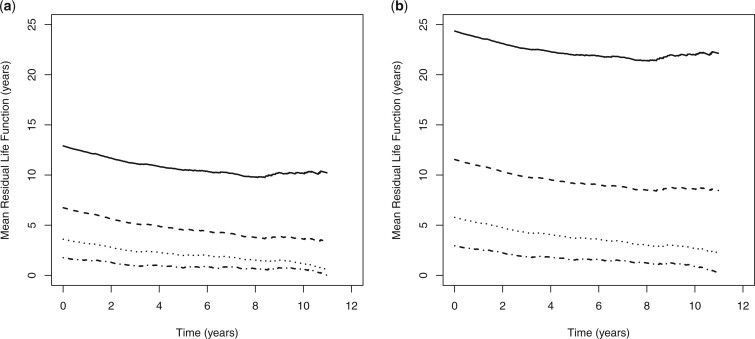
Predicted mean residual life functions by age for (a) male patients and (b) female patients, with zero subject effect (age at diagnosis: 50 years [solid line]; 60 years [dashed line]; 70 years [dotted line]; 80 years [dashed-dotted line]).

The predicted survival with respect to multimorbidity and death by age for male and female patients with zero frailty (subject effect) living in a major city or inner regional areas with melanoma site at head or neck, lesion thickness $>$ 1 mm, and ulceration are displayed in Figure 1. The corresponding MRL functions are given in Figure 2. It can be seen that the probability of survival (event free) is significantly higher for female patients and those diagnosed at a younger age. For example, around 50% of male patients aged 50 years at diagnosis with zero frailty will survive 10 years of event free for multimorbidity of a secondary cancer or death, whereas 70% of female patients aged 50 years will have the same survival period. The MRL functions shown in Figure 2 indicate slightly increasing hazard over time (constant hazard is observed after 8 years postdiagnosis for younger patients). Male patients diagnosed at the age of 50 years with zero frailty have roughly 13-year event-free period initially; those patients survived past 5 years postdiagnosis will have about 10.5 years event-free time. Again, there is evidence of significant age and gender differences. For example, male patients aged 60 years at diagnosis with zero frailty have multimorbidity of a secondary cancer or death approximately 6 years earlier compared with male patients diagnosed at the age of 50 years. Comparing patients diagnosed at the age of 50 years with zero frailty, females are event free for approximately 11.5 years more than males (see Figures 2(a and b)).

### 3.2. Simulation studies

Based on a cancer registry data structure, we generate ${\boldsymbol{X}}_j$, $T_{jk}^R$, $T_j^D$, and $S_j$ for $M=500$ patients as follows: (i) two-dimensional $(p=2)$ vector of ${\boldsymbol{X}}_j$: a binary covariate variable $X_{j1}$ is generated from Bernoulli distribution with a 0.5 probability, whereas a continuous covariate variable $X_{j2}$ is generated from a standard normal distribution N(0,1); (ii) Correlated gap times to recurrent events and death: multivariate random vector ${\boldsymbol{q}}$ is generated from the multivariate normal distribution $N({\boldsymbol{0}},\Gamma \otimes I_{M})$ given $\Gamma$ in (2.1) and parameters $\theta_{u}^{2}$, $\theta_{v}^{2}$ and $\rho$, then the gap times $T_{jk}^R$ and $T_j^D$ are generated using the cumulative hazard inversion method from the joint frailty model (2.2) given parameters ${\boldsymbol{\beta}}$ and ${\boldsymbol{\gamma}}$, where the baseline hazards are taken to be Weibull distributions with scale parameter $\lambda_i$ and shape parameter $\tau_i$ for $i=R$ or $i=D$; (iii) Administrative follow-up time $S_j$ is generated from a uniform distribution $U(a,b)$ representing a cancer registry with follow-up of at least $a$ days.

For each patient $j=(1,\ldots,M)$, the observed failure time $t_{jk}$ is obtained by $t_{jk}= \min(t_{jk}^R,t_j^D,C_j)$ repeatedly until $\sum_k t_{jk} \geq S_j$. The indicator variables $\delta_{jk}^R$ and $\delta_j^D$ are obtained according to the type of the event for $j=1,\ldots,M; \ k=1,\ldots,n_j$. We compare the proposed joint frailty model with informative censoring to the classical frailty model under eleven sets of scenario in a wide variety of contexts. Assessment is based on 500 replicated simulations for each set. Table 2 presents the comparison for the first six sets, in terms of the average bias, the average of the standard error estimates (SEE), the sample standard error of the estimates over 500 replications (SE), and the coverage probability (CP) of 95% confidence interval based on the normal approximation. Five additional simulated data sets, presented in Table S1 of Supplementary material available at *Biostatistics* online, assess the performance under a different setting of covariate effects and examine the robustness of the model to mis-specification of the normality assumption of the random effects.

**Table 2.** Results of simulated data (sets 1–6)

	Parameter (True value)	Joint frailty model				Frailty model$^\dagger$			
		Bias	SEE	SE	CP	Bias	SEE	SE	CP
Set 1	$\beta_1 \ (-0.6)$	0.029	0.37	0.36	0.96	0.060	0.36	0.36	0.95
	$\beta_2 \ (0.8)$	–0.034	0.19	0.19	0.93	–0.053	0.18	0.19	0.92
	$\gamma_1 \ (-0.8)$	0.012	0.25	0.24	0.97	0.081	0.24	0.23	0.96
	$\gamma_2 \ (0.5)$	0.019	0.13	0.12	0.96	–0.066	0.12	0.11	0.89
	$\theta_u \ (0.8)$	0.048	0.06	0.17	0.85	–0.146	0.73	0.27	1.00
	$\theta_v \ (0.8)$	0.030	0.06	0.11	0.88	–0.611	n.a.	0.18	n.a.
	$\rho \ (0.8)$	0.008	0.02	0.03	0.88		n.a.		
Set 2	$\beta_1 \ (-0.6)$	0.067	0.24	0.23	0.95	0.190	0.22	0.23	0.82
	$\beta_2 \ (0.8)$	–0.066	0.12	0.13	0.91	–0.140	0.12	0.12	0.75
	$\gamma_1 \ (-0.8)$	0.047	0.18	0.18	0.96	0.232	0.15	0.15	0.64
	$\gamma_2 \ (0.5)$	0.014	0.09	0.09	0.96	–0.199	0.07	0.07	0.25
	$\theta_u \ (1.5)$	–0.031	0.16	0.21	0.84	–0.700	0.30	0.20	0.32
	$\theta_v \ (1.5)$	–0.034	0.16	0.18	0.89	–1.269	n.a.	0.12	n.a.
	$\rho \ (0.9)$	0.003	0.01	0.01	0.89		n.a.		
Set 3	$\beta_1 \ (-0.6)$	0.054	0.22	0.22	0.95	0.247	0.20	0.21	0.75
	$\beta_2 \ (0.8)$	–0.060	0.12	0.12	0.91	–0.174	0.10	0.11	0.61
	$\gamma_1 \ (-0.8)$	0.054	0.18	0.18	0.94	0.301	0.13	0.13	0.35
	$\gamma_2 \ (0.5)$	0.013	0.09	0.09	0.95	–0.260	0.07	0.06	0.02
	$\theta_u \ (2.0)$	–0.051	0.27	0.32	0.86	–1.100	0.25	0.20	0.01
	$\theta_v \ (2.0)$	–0.021	0.28	0.31	0.90	–1.764	n.a.	0.11	n.a.
	$\rho \ (0.95)$	0.004	0.01	0.01	0.84		n.a.		
Set 4	$\beta_1 \ (-0.6)$	0.017	0.36	0.36	0.96	0.026	0.36	0.36	0.95
	$\beta_2 \ (0.8)$	–0.029	0.18	0.18	0.95	–0.034	0.18	0.18	0.94
	$\gamma_1 \ (-0.8)$	0.011	0.25	0.24	0.97	0.080	0.24	0.23	0.95
	$\gamma_2 \ (0.5)$	0.015	0.13	0.12	0.97	–0.065	0.12	0.12	0.92
	$\theta_u \ (0.8)$	0.030	0.06	0.15	0.89	–0.143	0.68	0.26	1.00
	$\theta_v \ (0.8)$	–0.001	0.06	0.04	0.99	–0.604	n.a.	0.17	n.a.
	$\rho \ (0.2)$	0.047	0.05	0.10	0.83		n.a.		
Set 5	$\beta_1 \ (-0.6)$	0.046	0.28	0.27	0.94	0.105	0.27	0.27	0.92
	$\beta_2 \ (0.8)$	–0.039	0.14	0.14	0.93	–0.074	0.14	0.14	0.90
	$\gamma_1 \ (-0.8)$	0.022	0.19	0.18	0.96	0.130	0.17	0.17	0.90
	$\gamma_2 \ (0.5)$	0.019	0.10	0.09	0.95	–0.109	0.09	0.09	0.77
	$\theta_u \ (0.8)$	0.044	0.07	0.13	0.89	–0.202	0.43	0.19	1.00
	$\theta_v \ (0.8)$	0.033	0.07	0.09	0.92	–0.589	n.a.	0.14	n.a.
	$\rho \ (0.8)$	0.009	0.02	0.02	0.89		n.a.		
Set 6	$\beta_1 \ (-0.6)$	0.002	0.26	0.25	0.95	0.031	0.25	0.25	0.96
	$\beta_2 \ (0.8)$	–0.037	0.13	0.12	0.96	–0.053	0.13	0.12	0.94
	$\gamma_1 \ (-0.8)$	0.014	0.18	0.18	0.95	0.085	0.17	0.17	0.91
	$\gamma_2 \ (0.5)$	0.010	0.09	0.09	0.95	–0.073	0.08	0.09	0.83
	$\theta_u \ (0.8)$	0.018	0.04	0.08	0.93	–0.170	0.51	0.20	1.00
	$\theta_v \ (0.8)$	0.011	0.04	0.05	0.94	–0.626	n.a.	0.14	n.a.
	$\rho \ (0.8)$	0.004	0.01	0.01	0.94		n.a.		

$^\dagger$ Standard frailty model applies separately to multimorbidity and death.

Set 1 in Table 2 is the base model. We consider Weibull distributions with a low start but increasing hazard ($\lambda_R=3 \times 10^{-6}$; $\lambda_D=2 \times 10^{-6}$; $\tau_R=1.3$; $\tau_D=1.5$) and moderate variance component parameters ($\theta_{u}=\theta_{v}=\rho=0.8$). Covariate effects are specified in ${\boldsymbol{\beta}}$ and ${\boldsymbol{\gamma}}$ such that the binary covariate variable $X_{j1}$ reduces failure risk of multimorbidity and death, whereas the continuous covariate variable $X_{j2}$ increases both failure risks. We take $a=1825$ and $b=2200$ for an administrative follow-up period of at least 5 years. The averaged censoring proportion of 500 replicated simulations for $\delta_j^D$ is 83.7%.

In Sets 2 and 3, we assess the performance of the models under the scenarios of increased variance component parameters ($\theta_{u}=\theta_{v}=1.5$ and $\rho=0.9$; $\theta_{u}=\theta_{v}=2$ and $\rho=0.95$, respectively; the latter corresponds to a setting close to that of the real melanoma data set in Table 1, where $\rho$ is close to 1). We also raise the hazard risks ($\tau_R=1.5$ and $\tau_D=1.7$; $\tau_R=1.6$ and $\tau_D=1.8$, respectively). The corresponding averaged censoring proportions for $\delta_j^D$ in Sets 2 and 3 are 57.3% and 43.8%, respectively.

In Set 4, we study the situation where the dependence between multimorbidity and death is small ($\rho=0.2$) while keeping all other parameters the same as those in the base model in Set 1. The corresponding averaged censoring proportion for $\delta_j^D$ in Set 4 is 83.6%. Next, we consider a longer follow-up period of at least 10 years by taking $a=3650$ and $b=4000$ in Set 5 and a large sample size ($M=1000$) in Set 6 (the averaged censoring proportion for $\delta_j^D$ are 68.5% and 83.8%, respectively). All other parameters are set as in the base model.

In Set 7 (Table S1 of Supplementary material available at *Biostatistics* online), we assess the performance under a different setting of covariate effects (here, both $X_{j1}$ and $X_{j2}$ increase the risk of multimorbidity, whereas $X_{j1}$ increases but $X_{j2}$ reduces the risk of death). In Set 8, we examine the robustness of the model to mis-specification of the normality assumption of the random effects by generating ${\boldsymbol{q}}$ from mixtures of two normal distributions. The corresponding averaged censoring proportions for $\delta_j^D$ in Sets 7 and 8 are 71.3% and 84.0%, respectively. Moreover, we consider a larger sample size ($M=2000$) in Set 9 to illustrate the asymptotic behavior and a setting with negative correlation (Set 10) as well as an independence setting with correlation $\rho=0$ (Set 11). The averaged censoring proportions for $\delta_j^D$ in Sets 9, 10, and 11 are 83.8%, 83.3%, and 83.5%, respectively.

From Table 2 and Table S1 of Supplementary material available at *Biostatistics* online, no appreciable bias is observed in all simulation settings, confirming the applicability of the proposed joint frailty model in a wide variety of contexts. In general, there is good agreement between SEE and SE for all the fixed-effect parameters, indicating that the standard errors of these parameters are well estimated. The SEE and SE are also comparable for the variance components, except $\theta_{u}$ for multimorbidity when the variance component parameters are small ($\le 0.8$ in all sets except Sets 2 and 4). This is also reflected in the CP, which is lower than the nominal level. This finding implies that the standard error of $\theta_{u}^{2}$ may be underestimated in some situations and thus caution should be exercised in interpreting the significance level to this variance component parameter; see Tawiah *and others* (2020a,b) for discussion on formal tests of heterogeneity when the prediction of subject-specific frailties is relevant. As expected, settings with a longer follow-up (Set 5) or larger sample sizes in Sets 6 and 9 lead to improved results (e.g., lower SE or better CP). Comparatively, the estimates obtained from separate standard frailty models have generally a larger bias. In particular, when the variance component parameters are large (Sets 2 and 3), the estimates of the fixed-effect and variance component parameters are heavily biased. In all settings, the standard errors of $\theta_{u}^{2}$ are overestimated.

## 4. Discussion

Multimorbidity is increasingly recognized as an important issue in health sciences, imposing serious challenges on the world’s healthcare systems. Modern study designs and data-linkage technologies have enabled the collection of longitudinal data that can advance our knowledge on the evolution of multimorbidity, which is valuable for developing interventions and care management strategies to better help a large number of individuals with multiple conditions. What is missing is an efficient modeling technique to overcome the methodological barriers in time-to-event data analyses, due to heterogeneous failure risk and the presence of a terminal event. In this article, we have developed a new joint frailty modeling framework within the GLMM for efficient estimation of model parameters in survival analysis of recurrent time-to-event data. This is timely given the heavy investments worldwide in conducting large-scale studies associated with multimorbidity.

Our approach retains the cancelation of baseline hazard property, which makes the estimation procedures relatively efficient. It also yields a more general model through the use of multivariate random effects to model jointly the dependence between the hazard rates of recurrent events and a terminal event. More importantly, the proposed joint frailty model provides clinically important information on event-free survival and residual life as well as prediction of patient-specific frailties for both recurrent and terminal events, beyond those that can be observed from existing frailty models. The applicability of the proposed method to advance our knowledge on the evolution of multimorbidity is illustrated using a real cancer registry data of melanoma patients in Australia, where multimorbidity is referred to as the occurrence of secondary primary cancer other than melanoma. We show how the failure risk of multimorbidity and death can be summarized using the predicted survival and MRL functions. The performance of the joint frailty model is demonstrated via simulation studies, which show its superiority over separate standard frailty models and its robustness in a wide variety of contexts (including the scenarios with zero, low, moderate, and high positive correlations as well as a negative correlation) and to the violation of normality assumption of random effects. In practice, there are a variety of choices of random effect distributions in GLMM, including gamma, normal (log-normal), t, Cauchy, or a mixture of discrete distributions (Masci *and others,* 2020). The results of joint frailty modeling of multivariate survival data should not be sensitive to the choice of the frailty distribution in general (Rondeau *and others,* 2007). In addition to the metrics considered, the discriminatory ability of the model can be assessed in terms of concordance c-index. Using the “survcomp” package for the melanoma data example, the c-index of 0.950 for the proposed joint frailty model for death is larger than the standard frailty model (c-index = 0.861), indicating its better performance in prediction in survival analysis. However, the c-indices here are for model comparison only, as Brentnall and Cuzick (2018) have shown that classical estimators of the c-index may be biased when there are converging hazards.

One important feature of the GLMM formulation involves the construction of a partial log-likelihood of failure times with the random effects conditionally fixed, as presented in $l_1$ of (2.4). Under mild conditions, the maximum partial likelihood estimation of the fixed covariate effects is unbiased and asymptotically normally distributed, with variances being estimated by the information matrix; see, for example, Yau (2001). Furthermore, the GLMM formulation has been generalized by Lee and Nelder (1996) and was named the hierarchical generalized linear model, where the normality assumption of the random effects is relaxed to allow for various families of distribution. Maximizing the log (hierarchical) h-likelihood $l$ gives fixed effect estimators that are asymptotically equivalent to those obtained from the use of marginal likelihood and hence inherits asymptotic consistency, efficiency, and normality of the GLMM. Recently, Lee and Kim (2020) studied the properties of the maximum h-likelihood estimators (MHLEs) for random effects in clustered data. They concluded that, as the number of clusters tends to infinity with a fixed cluster size, the MHLE for random effects can be obtained from the h-likelihood that is asymptotically valid in large samples; see also Lee *and others* (2017). However, to obtain asymptotically best-unbiased predictors for the random effects needs the condition that the number of random effects does not increase with the sample size, which is not always realistic. Our simulation studies do show improved results (lower SE or better CP) when sample size increases from Set 1 to Sets 6 and 9. Figure S1 of Supplementary material available at *Biostatistics* online presents the distributions of estimated fixed-effect parameters for Sets 1, 6, and 9. It shows that the distributions approach a normal distribution when the sample size increases.

In survival analysis of recurrent event data, there may exist a subgroup of patients who have recovered (or cured) and thus will not experience any failure event. In these situations, a joint frailty mixture cure model can be adopted to explain the patient-specific frailties that affect the cure proportion in the incidence component via logistic modeling (Tawiah *and others,* 2020). Justification of the use of cure models requires the evidence of the existence of long-term survivors, which is characterized by the survival curves being leveled at nonzero probabilities. The proposed joint frailty model is appropriate as the survival curves displayed in Figure 1 do not suggest the necessity of a cure model for analyzing the melanoma cancer registry data.

The proposed method can be readily applicable to solve health problems in the general context of multimorbidity, where time to multimorbidity corresponds to the occurrence of comorbid chronic diseases within an individual. To account for different disease types, the proposed joint frailty model may be extended to incorporate the types of chronic diseases in the modeling of recurrent events (see Beesley and Taylor, 2019). Preclassification of chronic diseases into a few multimorbidity “clusters” (Ng *and others,* 2018) can reduce the number of event types and hence the complexity of the joint frailty model so formed. Alternatively, multistate survival models may be adopted to account for competing events at each transition between disease states; see Crowther and Lambert (2017), Williams *and others* (2020) and the review by Hougaard (1999). This approach formulates multiple states via a stochastic process (e.g., Spreafico and Ieva (2020)) and emphasizes on modeling transition probabilities between states using Markov or semi-Markov models. Multistate survival models are particularly important in studies where covariate effects for each specific transition between disease states are the primary interest. The complexity of multistate models for recurrent events increases with the numbers of states and recurrences. In some situations, model simplification (e.g., assumption of constant transition hazards) is required to make the multistate approach work. The integration of the multistate approach into the joint frailty model, along with other model extensions such as the accelerated failure time or “interval censoring” assumptions, will be pursued in future research.

## Supplementary Material

kxab037_Supplementary_Data

## 5. Software

R code used in this article is available on GitHub (https://github.com/RichardTawiah/JointFrailty) and is available on request from the corresponding author (s.ng@griffith.edu.au).
