# Supplementary material for: Joint frailty modeling of time-to-event data to elicit the evolution pathway of events: a generalized linear mixed model approach
Source: Biostatistics. 2021 Nov 9;24(1):108–23. doi: 10.1093/biostatistics/kxab037 (PMC9766887; doi:10.1093/biostatistics/kxab037)
Supplement: kxab037_Supplementary_Data [file kxab037_supplementary_data.zip › kxab037_Supplementary_Data/Biostatistics_Supplementary_Material.pdf]

Biostatistics (2021), **0**, 0, *pp.* 1–7
doi:10.1093/biostatistics/output

Joint frailty modelling of time-to-event data to elicit the evolution pathway of events: A generalised linear mixed model approach

Supplementary Materials

SHU-KAY NG*

School of Medicine and Dentistry, Menzies Health Institute Queensland, Griffith University, Australia

s.ng@griffith.edu.au

RICHARD TAWIAH

School of Mathematics and Statistics, University of Melbourne, Australia

GEOFFREY J. MCLACHLAN

Department of Mathematics, University of Queensland, Australia

VINOD GOPALAN

School of Medicine and Dentistry, Menzies Health Institute Queensland, Griffith University, Australia

S1. Estimation of variance components and asymptotic variances

The proposed joint modelling method adopts a GLMM approach within a frailty modelling framework to account for correlation among individual times to (recurrent) events and death.

*To whom correspondence should be addressed.

© The Author 2021. Published by Oxford University Press. All rights reserved. For permissions, please e-mail: journals.permissions@oup.com

As illustrated in the Methods section, the GLMM method involves the computations of the first derivatives of l_1 with respect to η and ζ within the Newton-Raphson iterative procedure in Equation (2.6) in the main text. Let $Q_1 = \text{diag}(\varpi_1^R, ..., \varpi_N^R)$, $E_1 = \text{diag}(e_1^R, ..., e_N^R)$, and $S_1 = \text{diag}(s_1^R, ..., S_N^R)$, where $\varpi_l^R = \exp(\eta_l)$, $e_l^R = \delta_l^R / \sum_{j=1}^N \varpi_j^R$, and $s_l^R = \sum_{j=1}^l e_j^R$ $(l = 1, \ldots, N)$. It can be shown that

$$\frac{\partial l_1}{\partial \eta} = \Delta^R - Q_1 F_1 E_1 \mathbf{1}_N$$

and

$$-\frac{\partial^2 l_1}{\partial \eta \partial \eta^T} = Q_1 S_1 - Q_1 F_1 E_1^2 F_1^T Q_1,$$

where Δ^R is the vector of censoring indicators for the gap times between recurrent events, F_1 is a $N \times N$ lower-triangular matrix with non-zero elements of one, and $\mathbf{1}_N$ is a vector of ones with dimension being specified by the subscript. Similarly, we have

$$\frac{\partial l_1}{\partial \zeta} = \Delta^D - Q_2 F_2 E_2 \mathbf{1}_M$$

and

$$-\frac{\partial^2 l_1}{\partial \zeta \partial \zeta^T} = Q_2 S_2 - Q_2 F_2 E_2^2 F_2^T Q_2,$$

where Δ^D is the vector of censoring indicators for the death time; E_2, F_2, Q_2, and S_2 have the same form as E_1, F_1, Q_1, and S_1, respectively, with a dimension of M instead of N. The information matrix $\boldsymbol{G}$ in Section 2.2 of the main text is a simplification of $-\partial^2 l_\Omega / \partial\Omega\partial\Omega^T$, given by

$$\boldsymbol{G} = \begin{bmatrix} X_1^T & 0 \\ 0 & X_2^T \\ Z_1^T & 0 \\ 0 & Z_2^T \end{bmatrix} \begin{bmatrix} D_{\eta\eta} & D_{\eta\zeta} \\ D_{\zeta\eta} & D_{\zeta\zeta} \end{bmatrix} \quad \begin{bmatrix} X_1 & 0 & Z_1 & 0 \\ 0 & X_2 & 0 & Z_2 \end{bmatrix} +$$

$$\frac{1}{\theta_u^2 \theta_v^2 (1-\rho^2)} \begin{bmatrix} 0 & 0 & 0 & 0 \\ 0 & 0 & 0 & 0 \\ 0 & 0 & \theta_v^2 I_M & -\rho\theta_u\theta_v I_M \\ 0 & 0 & -\rho\theta_u\theta_v I_M & \theta_u^2 \end{bmatrix},$$

where $D_{\eta\eta} = -\partial^2 l_1/\partial\eta\partial\eta^T$, $D_{\zeta\zeta} = -\partial^2 l_1/\partial\zeta\partial\zeta^T$ and $D_{\eta\zeta} = D_{\zeta\eta} = -\partial^2 l_1/\partial\eta\partial\zeta^T = -\partial^2 l_1/\partial\zeta\partial\eta^T = 0$. It follows that the inverse matrix of $\boldsymbol{G}$ can be written in a block form as:

$$\boldsymbol{G}^{-1} = \begin{bmatrix} \boldsymbol{B}_{\beta,\beta} & \boldsymbol{B}_{\beta,\gamma} & \boldsymbol{B}_{\beta,q} \\ \boldsymbol{B}_{\gamma,\beta} & \boldsymbol{B}_{\gamma,\gamma} & \boldsymbol{B}_{\gamma,q} \\ \boldsymbol{B}_{q,\beta} & \boldsymbol{B}_{q,\gamma} & \boldsymbol{B}_{q,q} \end{bmatrix},$$

where the asymptotic standard errors of $\boldsymbol{\beta}$ and $\boldsymbol{\gamma}$ are given by

$$\text{var}\begin{bmatrix} \hat{\boldsymbol{\beta}} \\ \hat{\boldsymbol{\gamma}} \end{bmatrix} = \begin{bmatrix} \boldsymbol{B}_{\beta,\beta} & \boldsymbol{B}_{\beta,\gamma} \\ \boldsymbol{B}_{\gamma,\beta} & \boldsymbol{B}_{\gamma,\gamma} \end{bmatrix}. \tag{S1.1}$$

For the variance component parameters $\boldsymbol{\Phi} = (\theta_u^2, \theta_v^2, \rho)^T$, the equation of the first order derivative of the REML log likelihood is

$$\text{tr}\boldsymbol{\Sigma}^{-1}\frac{\partial\boldsymbol{\Sigma}}{\partial\boldsymbol{\Phi}} + \text{tr}\left(\boldsymbol{B}_{q,q} + \boldsymbol{q}\boldsymbol{q}^T\right)\frac{\partial\boldsymbol{\Sigma}^{-1}}{\partial\boldsymbol{\Phi}} = 0, \tag{S1.2}$$

where, tr denotes the trace of a matrix. Solving (S1.2), we have

$$\text{tr}\boldsymbol{\Sigma}^{-1}\frac{\partial\boldsymbol{\Sigma}}{\partial\theta_u^2} = \frac{M}{\theta_u}, \quad \text{tr}\boldsymbol{\Sigma}^{-1}\frac{\partial\boldsymbol{\Sigma}}{\partial\theta_v^2} = \frac{M}{\theta_v}, \quad \text{tr}\boldsymbol{\Sigma}^{-1}\frac{\partial\boldsymbol{\Sigma}}{\partial\rho} = -\frac{2M\rho}{(1-\rho^2)}. \tag{S1.3}$$

and that

$$\begin{aligned} \text{tr}(\boldsymbol{B}_{q,q} + \boldsymbol{q}\boldsymbol{q}^T)\frac{\partial\boldsymbol{\Sigma}^{-1}}{\partial\theta_u^2} &= \frac{1}{\theta_u^4\theta_v^2(1-\rho^2)}(-\theta_v^2\Im_1 + \rho\theta_u\theta_v\Im_2), \\ \text{tr}(\boldsymbol{B}_{q,q} + \boldsymbol{q}\boldsymbol{q}^T)\frac{\partial\boldsymbol{\Sigma}^{-1}}{\partial\theta_v^2} &= \frac{1}{\theta_u^2\theta_v^4(1-\rho^2)}(-\theta_u^2\Im_3 + \rho\theta_u\theta_v\Im_2), \\ \text{tr}(\boldsymbol{B}_{q,q} + \boldsymbol{q}\boldsymbol{q}^T)\frac{\partial\boldsymbol{\Sigma}^{-1}}{\partial\rho} &= \frac{1}{\theta_u^2\theta_v^2(1-\rho^2)^2}(-2\rho\theta_v^2\Im_1 - 2(1+\rho)\theta_u\theta_v\Im_2 + 2\rho\theta_u^2\Im_3), \end{aligned} \tag{S1.4}$$

where $\Im_1 = \text{tr}\left\{K_1\left(\boldsymbol{B}_{q,q} + \boldsymbol{q}\boldsymbol{q}^T\right)\right\}$, $\Im_2 = \text{tr}\left\{K_2\left(\boldsymbol{B}_{q,q} + \boldsymbol{q}\boldsymbol{q}^T\right)\right\}/2$, $\Im_3 = \text{tr}\left\{K_3\left(\boldsymbol{B}_{q,q} + \boldsymbol{q}\boldsymbol{q}^T\right)\right\}$ and

$$K_1 = \begin{bmatrix} I_M & 0 \\ 0 & 0 \end{bmatrix}, \quad K_2 = \begin{bmatrix} 0 & I_M \\ I_M & 0 \end{bmatrix}, \quad \text{and} \quad K_3 = \begin{bmatrix} 0 & 0 \\ 0 & I_M \end{bmatrix}.$$

Further simplifying the resulting equations by substituting Equations (S1.3) and (S1.4) into (S1.2), we obtain the REML estimators for θ_u^2, θ_v^2 and ρ given in Equation (2.7) in the main text.

The asymptotic standard errors of θ_u^2, θ_v^2, and ρ are given by:

$$\text{var}\begin{bmatrix} \hat{\theta}_u^2 \\ \hat{\theta}_v^2 \\ \hat{\rho} \end{bmatrix} = 2\begin{bmatrix} a_{11} & a_{12} & a_{13} \\ {a_{12}}^T & a_{22} & a_{23} \\ {a_{13}}^T & {a_{23}}^T & a_{33} \end{bmatrix}^{-1}, \tag{S1.5}$$

where

$$a_{11} = \mathrm{tr}(J_1 - J_2)^2, \quad a_{12} = \mathrm{tr}(J_1 J_3 + J_2 J_4 - 2J_1 J_4),$$

$$a_{13} = \mathrm{tr}(J_1 J_5 + J_2 J_6 - 2J_1 J_6), \quad a_{22} = \mathrm{tr}(J_3 - J_4)^4,$$

$$a_{23} = \mathrm{tr}(J_3 J_5 + J_4 J_6 - 2J_3 J_6), \quad a_{33} = \mathrm{tr}(J_5 - J_6)^2,$$

and where

$$J_1 = \boldsymbol{B}_{q,q} \frac{\partial \boldsymbol{\Sigma}^{-1}}{\partial \theta_u^2}, J_2 = \boldsymbol{\Sigma} \frac{\partial \boldsymbol{\Sigma}^{-1}}{\partial \theta_u^2}, J_3 = \boldsymbol{B}_{q,q} \frac{\partial \boldsymbol{\Sigma}^{-1}}{\partial \theta_v^2},$$

$$J_4 = \boldsymbol{\Sigma} \frac{\partial \boldsymbol{\Sigma}^{-1}}{\partial \theta_v^2}, J_5 = \boldsymbol{B}_{q,q} \frac{\partial \boldsymbol{\Sigma}^{-1}}{\partial \rho}, J_6 = \boldsymbol{\Sigma} \frac{\partial \boldsymbol{\Sigma}^{-1}}{\partial \rho}.$$

S2. Additional simulation data sets

As described in the Results section of the main text, five additional simulated data sets were generated. In Set 7, we assess the performance under a different setting of covariate effects (here, both X_{j1} and X_{j2} increase the risk of multimorbidity, whereas X_{j1} increases but X_{j2} reduces the risk of death). In Set 8, we examine the robustness of the model to mis-specification of the normality assumption of the random effects by generating $\boldsymbol{q}$ from mixtures of two normal distributions. In Set 9, we consider a larger sample size ($M = 2000$) to illustrate the asymptotic behaviour. We also examine a setting with negative correlation in Set 10 as well as an independence setting with correlation $\rho = 0$ in Set 11. All other parameters were kept the same as those in the base model in Set 1.

We compare the proposed joint frailty model with informative censoring to a standard frailty model separately for multimorbidity and death events. Assessment is based on 500 replicated simulations for each set. The corresponding averaged censoring proportions for δ_j^D in Sets 7 and 8 are 71.3% and 84.0%, respectively. In Sets 9, 10, and 11, the averaged censoring proportions for δ_j^D are 83.8%, 83.3%, and 83.5%, respectively. Table S1 presents the comparison for Sets 7 to 11, in terms of the average bias, the average of the standard error estimates (SEE), the sample

standard error of the estimates over 500 replications (SE), and the coverage probability (CP) of 95% confidence interval based on the normal approximation. From Table S1, no appreciable bias is observed, confirming the applicability of the proposed joint frailty model in these additional settings. In general, there is good agreement between SEE and SE for all the fixed-effect parameters, indicating that the standard errors of these parameters are well estimated. The SEE and SE are also comparable for the variance components, except θ_u for multimorbidity when the variance component parameters are small ($\leqslant 0.8$). This is also reflected in the CP, which is lower than the nominal level. This finding implies that the standard error of θ_u^2 may be underestimated in some situations and thus caution should be exercised in interpreting the significance level to this variance component parameter; see the main text for discussion on formal tests of heterogeneity when prediction of subject-specific frailties is relevant. Comparatively, the estimates obtained from separate standard frailty models have generally a larger bias. In these settings, the standard errors of θ_u^2 are overestimated using a standard frailty model.

As described in the Discussion section of the main text, we present the distributions of the estimates of fixed-effect parameters $\boldsymbol{\beta}$ and $\boldsymbol{\gamma}$ for Set 1 ($M = 500$), Set 6 ($M = 1000$), and Set 9 ($M = 2000$) in Figure S1, overlaid with standard normal curves. The normality test results using Shapiro-Wilk's method show that the distributions approach a normal distribution when the sample size M increases.

Table S1. *Results of simulated data (Sets 7 to 11)*

	Parameter (True value)	Joint frailty model				§Frailty model			
		Bias	SEE	SE	CP	Bias	SEE	SE	CP
Set 7	β_1 (0.3)	-0.019	0.33	0.32	0.95	-0.062	0.32	0.32	0.95
	β_2 (0.5)	-0.009	0.16	0.16	0.96	0.009	0.16	0.16	0.96
	γ_1 (0.8)	-0.021	0.20	0.18	0.97	-0.089	0.18	0.17	0.94
	γ_2 (-0.3)	0.038	0.10	0.10	0.94	0.018	0.09	0.09	0.95
	θ_u (0.8)	0.042	0.07	0.16	0.85	-0.140	0.65	0.21	1.00
	θ_v (0.8)	0.015	0.07	0.09	0.91	-0.679	n.a.	0.12	n.a.
	ρ (0.8)	0.007	0.02	0.03	0.87		n.a.		
Set 8	β_1 (-0.6)	0.030	0.37	0.38	0.96	0.060	0.37	0.38	0.96
	β_2 (0.8)	-0.035	0.19	0.19	0.96	-0.052	0.18	0.19	0.95
	γ_1 (-0.8)	-0.003	0.26	0.25	0.96	0.065	0.24	0.24	0.95
	γ_2 (0.5)	0.009	0.13	0.12	0.96	-0.074	0.12	0.12	0.89
	θ_u (0.8)	0.038	0.06	0.14	0.88	-0.190	0.74	0.25	1.00
	θ_v (0.8)	0.025	0.06	0.10	0.91	-0.620	n.a.	0.17	n.a.
	ρ (0.8)	0.007	0.01	0.02	0.88		n.a.		
Set 9	β_1 (-0.6)	0.023	0.18	0.17	0.96	0.053	0.18	0.18	0.95
	β_2 (0.8)	-0.034	0.09	0.09	0.95	-0.052	0.09	0.09	0.91
	γ_1 (-0.8)	0.017	0.13	0.12	0.97	0.087	0.12	0.11	0.88
	γ_2 (0.5)	0.009	0.06	0.06	0.95	-0.074	0.06	0.06	0.75
	θ_u (0.8)	0.003	0.03	0.02	0.99	-0.109	0.36	0.11	1.00
	θ_v (0.8)	0.002	0.03	0.02	0.99	-0.609	n.a.	0.12	n.a.
	ρ (0.8)	0.001	0.01	0.01	0.99		n.a.		
Set 10	β_1 (-0.6)	0.012	0.35	0.36	0.94	-0.031	0.35	0.36	0.94
	β_2 (0.8)	-0.035	0.18	0.17	0.95	-0.008	0.18	0.17	0.96
	γ_1 (-0.8)	0.014	0.25	0.24	0.97	0.066	0.24	0.23	0.95
	γ_2 (0.5)	0.002	0.13	0.12	0.96	-0.053	0.12	0.11	0.92
	θ_u (0.8)	-0.029	0.06	0.05	0.89	-0.137	0.65	0.20	1.00
	θ_v (0.8)	-0.028	0.06	0.04	0.90	-0.609	n.a.	0.17	n.a.
	ρ (-0.8)	0.008	0.02	0.01	0.96		n.a.		
Set 11	β_1 (-0.6)	0.001	0.36	0.38	0.95	0.002	0.35	0.38	0.95
	β_2 (0.8)	-0.020	0.18	0.19	0.93	-0.020	0.18	0.19	0.92
	γ_1 (-0.8)	0.005	0.25	0.24	0.97	0.072	0.24	0.24	0.95
	γ_2 (0.5)	0.016	0.13	0.12	0.97	-0.061	0.12	0.11	0.92
	θ_u (0.8)	0.009	0.05	0.13	0.86	-0.147	0.67	0.26	1.00
	θ_v (0.8)	-0.019	0.06	0.04	0.95	-0.607	n.a.	0.18	n.a.
	ρ (0)	0.071	0.05	0.14	0.80		n.a.		

§ Standard frailty model applies separately to multimorbidity and death.

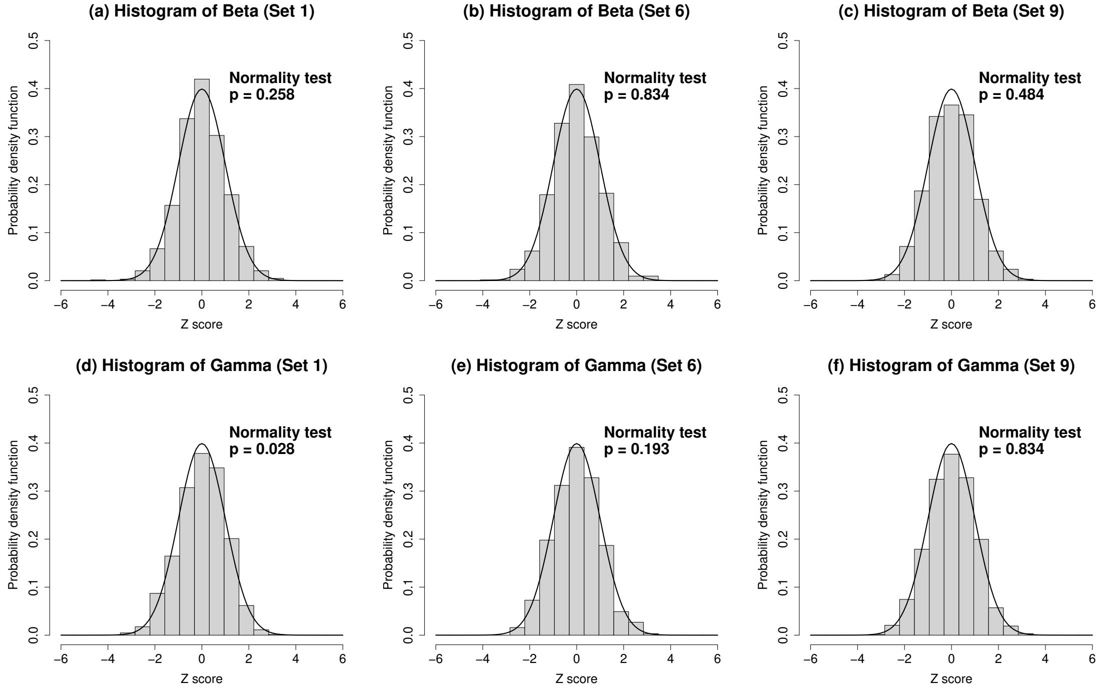

Fig. S1. Histogram of standardised estimates of fixed-effect parameters Beta and Gamma for Set 1 ($M = 500$), Set 6 ($M = 1000$), and Set 9 ($M = 2000$), overlaid with standard normal curves.
